# Burden of onchocerciasis-associated epilepsy: first estimates and research priorities

**DOI:** 10.1186/s40249-018-0481-9

**Published:** 2018-09-19

**Authors:** Natalie V. S. Vinkeles Melchers, Sarah Mollenkopf, Robert Colebunders, Michael Edlinger, Luc E. Coffeng, Julia Irani, Trésor Zola, Joseph N. Siewe, Sake J. de Vlas, Andrea S. Winkler, Wilma A. Stolk

**Affiliations:** 1 Department of Public Health, Erasmus MC, University Medical Center Rotterdam, P.O. box 2040, 3000 CA Rotterdam, The Netherlands; 2 Institute for Health Metrics and Evaluation, University of Washington, 2301 5th Avenue, Suite 600, Seattle, WA 98121 USA; 3 Global Health Institute, University of Antwerp, Antwerp, Belgium; 4 Department of Medical Statistics, Informatics, and Health Economics, Medical University Innsbruck, Vienna, Austria; 5 Department of Public Health, Institute of Tropical Medicine Antwerp, Nationalestraat 155, 2000 Antwerp, Belgium; 6 Department of Tropical Medicine, University of Kinshasa, Kinshasa, Democratic Republic of the Congo; 7 Centre for Global Health, Institute for Health and Society, Oslo, Norway; 8 Center for Global Health, Department of Neurology, Technical University of Munich, Munich, Germany

**Keywords:** River blindness, Onchocerciasis, Epilepsy, Burden estimates, Years of life lived with disability, Review, Research priorities, Prevalence, Disability weight, Case definition

## Abstract

**Background:**

Since the 1990s, evidence has accumulated of an increased prevalence of epilepsy in onchocerciasis-endemic areas in Africa as compared to onchocerciasis-free areas. Although the causal relationship between onchocerciasis and epilepsy has yet to be proven, there is likely an association. Here we discuss the need for disease burden estimates of onchocerciasis-associated epilepsy (OAE), provide them, detail how such estimates should be refined, and discuss the socioeconomic impact of OAE, including a cost-estimate for anti-epileptic drugs.

**Main body:**

Providing OAE burden estimates may aid prevention of epilepsy in onchocerciasis- endemic areas by inciting and informing collaboration between onchocerciasis control programmes and mental health services. Epilepsy not only massively impacts the health of those affected, but it also carries a high socioeconomic burden for the households and communities involved. We used previously published geospatial estimates of onchocerciasis in Africa and a separately published logistic regression model quantifying the association between onchocerciasis and epilepsy to estimate the number of OAE cases. We then applied disability weights for epilepsy to quantify the burden in terms of years of life lived with disability (YLD) and estimate the cost of treatment. We estimate that in 2015 roughly 117 000 people were affected by OAE across onchocerciasis-endemic areas previously under the African Programme for Onchocerciases control (APOC) mandate where OAE has ever been reported or suspected, and another 264 000 persons in onchocerciasis-endemic areas where OAE has never been investigated before. The total number of YLDs due to OAE was 39 300 and 88 700 in these areas respectively, based on a weighted mean disability weight of 0.336. The burden of OAE is approximately 13% of the total YLDs attributable to onchocerciasis and 10% of total YLDs attributable to epilepsy. We estimated that by 2015 the total costs of treatment with anti-epileptic drug for OAE cases would have been a minimum of 12.4 million US$.

**Conclusions:**

These estimates suggest a considerable health, social and economic burden of OAE in Africa. The treatment and care for people with epilepsy, especially in hyperendemic onchocerciasis areas with high epilepsy prevalence thus requires more financial and human resources.

**Electronic supplementary material:**

The online version of this article (10.1186/s40249-018-0481-9) contains supplementary material, which is available to authorized users.

## Multilingual abstracts

Please see Additional file 1 for translations of the abstract into the six official working languages of the United Nations.

## Background

Onchocerciasis, or “river blindness”, is targeted for elimination, using preventive chemotherapy through mass drug administration (MDA) with ivermectin as the primary intervention strategy [1]. Onchocerciasis is transmitted by the bite of infected blackflies that breed in fast-flowing rivers. It causes stigmatising skin disease and vision loss, the latter eventually leading to blindness, nearly all cases occurring in sub-Saharan Africa (SSA). Since the 1990s, high prevalence of epilepsy in onchocerciasis highly-endemic areas has increasingly been reported, especially in localised foci across Africa [2–8].

In general, the prevalence of epilepsy in sub-Saharan Africa is higher as compared to Asia, Europe and North America [9]; the mean prevalence in Africa is 26% higher than the global mean [10]. Epilepsy is more common in Africa due to several factors, including socioeconomic deprivations, limited access to high quality and affordable healthcare facilities, particularly in rural areas [10]. The Global Burden of Disease (GBD) study estimated for the year 2015 a total of 2.66 million disability-adjusted life years (DALYs) (95% *CI*: 2.15–3.28) attributed to epilepsy, and 0.99 million DALYs (95% *CI*: 0.45–1.72) attributable to onchocerciasis in SSA [11]. Various studies have estimated the number of people with active epilepsy in SSA with numbers ranging from 2.5 million to 4.5 million [10–12]. Only a fraction of these epilepsy cases may potentially be attributed to onchocerciasis-associated epilepsy (OAE) [13]. An early, crude assessment of the burden of OAE in SSA estimated approximately 100 000 cases (2011 data) [14]. Given the negative consequences of OAE, this number should be refined with more granular data and more advanced methods since these numbers were estimated as a proportion of a predicted number of *Onchocerca volvulus*-infected people in the absence of MDA. OAE-affected individuals are subject to high economic costs, stigmatisation, discrimination [15] and premature mortality [16] if left untreated.

In this review, we discuss the current evidence of an association between onchocerciasis and epilepsy, and provide the first estimates of OAE burden in terms of expected number of cases, years of life lived with disability (YLDs), and socioeconomic consequences for onchocerciasis-endemic areas previously under the African Programme for Onchocerciasis Control (APOC) mandate. Furthermore, we suggest research priorities to assist in building consensus on the prioritisation of the OAE research agenda and the diligence of human and financial resources required to prevent new OAE cases.

## Are onchocerciasis and epilepsy associated?

Many well-known, non-infectious causes of epilepsy may contribute to the burden of epilepsy in onchocerciasis-endemic areas, including perinatal trauma, genetic factors, environmental/toxic factors or nutritional deficiencies that occur early in life [9]. Some parasitic infections are known to be associated with epilepsy, including neurocysticercosis (NCC) (due to *Taenia solium*), toxoplasmosis (due to *Toxoplasma gondii*), and malaria, among others [9]. For example, *T. solium* in particular is endemic in many African countries where widespread free-roaming of pigs occurs and where pork is consumed [17], and it is estimated that around 30% of the acquired epilepsy in *T. solium*-endemic areas of developing countries is caused by NCC [18]. It is likely that NCC plays an important role in SSA, although there is little knowledge on how widespread the distribution of NCC in SSA is [17]. The role of other parasitic infections in causing epilepsy, including *O. volvulus* infection, has been much less established. Although several cross-sectional and case-control studies show an association between onchocerciasis and epilepsy [3, 4, 19, 20], it is challenging to interpret such studies and demonstrate causality in this association due to co-infection with multiple other parasites (e.g. *Plasmodium falciparum*, *T. gondii*, *T. solium* [21]) and other confounding factors.

On the population-level, there is evidence of an association between epilepsy and onchocerciasis. A meta-analysis by Pion et al. [4] found an association between onchocerciasis and epilepsy using population-based surveys; on average there was a 0.4% increase in epilepsy for each 10% increase in onchocerciasis prevalence. This association is based on studies from eight communities in seven African countries. In only two areas (in Cameroon) NCC was reported to be endemic [2, 4, 22], but additional information from one of these areas show that a maximum of four possible or borderline *T. solium*-infected individuals were found out of 53 people with epilepsy [5]. It should be noted, however, that detection of NCC could be missed as diagnosis on the basis of serologic tests alone would be incomplete due to low sensitivity or specificity [23, 24]. Another review performed a restricted analysis on case-control studies that controlled for gender, age and place of residence [3]. This review by Kaiser et al. found a weak positive association between skin snip positivity and epilepsy (pooled *OR* = 1.29; 95% *CI*: 0.93–1.79, *P* = 0.139). Additionally, it found that quantitative measures of infection intensity in individuals (i.e. mean microfilariae (mf), number of palpated nodules) was significantly higher in people with epilepsy (PWE) than in people without epilepsy (PWOE). In addition, preliminary results of a recent prospective study performed in the Mbam valley of Cameroon, looking at the incidence of epilepsy in *O. volvulus*-infected children at baseline in 1991–1993 with a follow-up in 2017, suggest that the incidence rate ratio of epilepsy was significantly higher in children with very high initial mf intensities/skin snips [25]. These results suggest a dose-response relationship wherein the risk of developing epilepsy in onchocerciasis patients is higher with increasing *O. volvulus* mf density, supporting the hypothesis that a proportion of epilepsy cases in an onchocerciasis-endemic area are to be caused by onchocerciasis. The effect of ivermectin on preventing new OAE cases or on reducing the seizure frequency of prevalent epilepsy cases is to be further investigated, although recent studies suggest that ivermectin has a positive effect on epilepsy incidence [26, 27]. It is also reported that ivermectin can reduce severity and frequency of epileptic seizures [28], but it is yet unclear if this is due to the anticonvulsant properties of ivermectin or due to flaws in the methodology of the respective study. More studies are currently underway to assess the impact of MDA on OAE [29].

There is still no definitive pathophysiologic explanation for the link between onchocerciasis and epilepsy. Studies in children with nodding syndrome (a childhood epilepsy disorder described in *O. volvulus-*endemic areas) suggest that antibodies to a protein (leiomodin-1) present in neurons may cross-react with a similar protein that is present in the parasite *O. volvulus* [30]. Further research herein would be strongly recommended.

## The challenges of defining an onchocerciasis-associated epilepsy case

In spite of the population-level association between onchocerciasis and epilepsy, it is difficult to attribute individual epilepsy cases to onchocerciasis. Epilepsy is a condition characterised by recurrent (two or more) afebrile epileptic seizures at least 24 h apart, unprovoked by any immediate identified cause, thus not due to an acute intracranial or extracranial condition [31]. Individuals with one unprovoked seizure but with a > 60% recurrence risk of epileptic seizures due to an enduring epileptogenic abnormality are also considered to be epileptic [31]. Whether an epileptic seizure associated with *O. volvulus* infection also has a > 60% chance of recurrence is unknown and may depend on the mf load and whether the person has been treated with ivermectin. Nonetheless, the chances of epilepsy being caused by onchocerciasis are more likely in areas with high onchocerciasis transmission rates, evidence of *O. volvulus* infection, and onset of epilepsy at young age (~ 5–18 years old) [32]. Exclusion of other causes leading to epilepsy, such as NCC, is often not optimal in rural settings due to the unavailability of neuroimaging and requires the establishment of an epilepsy-triaging system [33]. Without the ability to exclude all other causes of epilepsy, it is impossible to confirm a case as OAE. Proper differentiation between causes of epilepsy in remote areas across SSA, keeping the limited access to advanced technological instruments in mind, is still an area that should receive further attention. Studies investigating the prevalence of OAE should therefore always attempt to include a thorough medical/neurological history and examination as well as diagnosis of various parasitic infections, including NCC, malaria, and toxoplasmosis, among others.

## Quantifying the number of OAE cases in sub-Saharan Africa

In order to estimate the potential burden of OAE in Africa, we first identified areas where OAE has been reported or suspected (independent on whether the study found a significant association between onchocerciasis and epilepsy). We identified 19 areas in nine countries across SSA; Uganda [5, 26, 34, 35], Tanzania [36, 37], Cameroon [2], Nigeria [19], Central African Republic [20], Burundi [22], Benin [38], the Democratic Republic of Congo [39], and South Sudan [40]. Little knowledge is available from countries previously under the Onchocerciasis Control Programme (OCP)-mandate, but we expect negligible levels of probable OAE cases due to the long duration of vector control and MDA (OCP: 1974–2002), including in Benin [38]. We therefore focussed on areas previously under the APOC-mandate (“APOC-areas/countries”). For each APOC-area, population density data for 1995 was obtained using the APOC census (for more information, please be referred to the note of Additional file 2).

We first estimated the number of prevalent OAE cases prior to initiation of MDA with ivermectin (gradually introduced in the region since 1995, with exception of Kaduna, Nigeria (1991)). This was done by linking a previously published functional relationship between the pre-control community-level prevalence of infection and epilepsy [4] (corrected for background prevalence of epilepsy in settings with zero infection prevalence) to published estimates of the pre-control epidemiologically mapped distribution of infection prevalence in 20 APOC countries [41]. Details of the approach and the underlying assumptions are described in Table 1.

**Table 1:** Methods for calculating onchocerciasis-associated epilepsy (OAE) cases in the African Programme for Onchocerciasis Control (APOC) countries in 1995 (pre-control) and in 2015

**Fig. 1:**
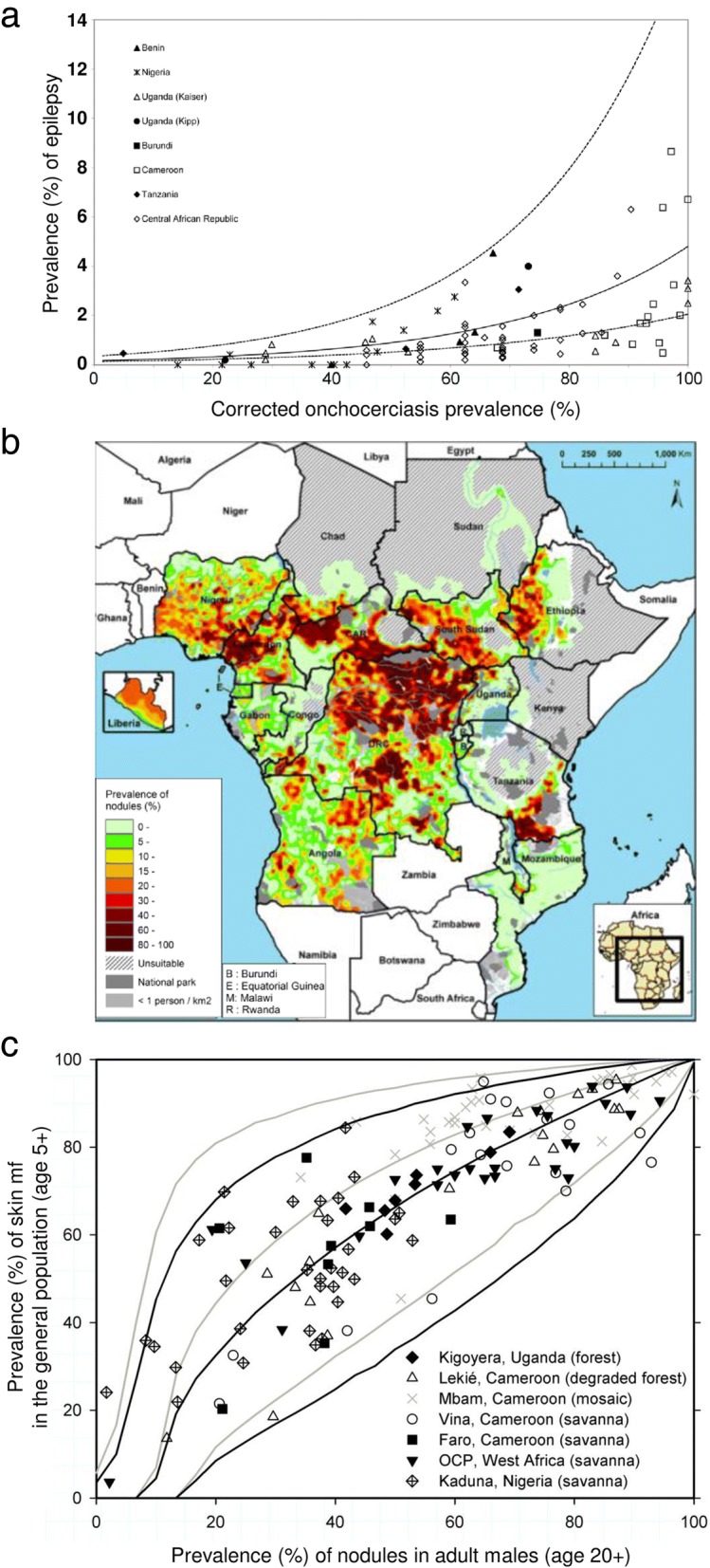
Used published relationships and onchocerciasis map to calculate the pre-control prevalence of onchocerciasis-associated epilepsy. **a** Community-level all-cause epilepsy prevalence versus corrected onchocerciasis microfilariae prevalence, as published by Pion et al. [4]. **b** Map of the estimated pre-control prevalence of palpable nodules in the 20 African Programme for Onchocerciasis Control countries, as published by Zouré et al. [41]. **c** Predicted skin mf prevalence in the general population, given observed nodule prevalence in adult males, as published by Coffeng et al. [69]. Permission for publication of all figures was granted from the journals and authors

Figure 1a shows the functional relationship describing the community-level association between the prevalence of *Onchocerca volvulus* skin microfilariae and all-cause epilepsy (case definition as in the International League Against Epilepsy guidelines [31]), as published by Pion et al. [4]. The predicted prevalence of epilepsy in areas with zero *O. volvulus* microfilariae prevalence was removed from the analysis. The prevalence of OAE in onchocerciasis-endemic areas was calculated by subtracting the predicted prevalence of all-cause epilepsy for APOC-areas using the functional relationship from an averaged all-cause background epilepsy prevalence for Sub-Saharan Africa (0.36%, 95% *CI*: 0.26–0.47% [11]). We linked the functional relationship to a published map of nodule prevalence in adult males in Africa (Fig. 1b) after converting this map to skin microfilariae prevalence in the general population (age 5 and above) at the pixel level (1 × 1 km raster) using a published statistical model (Fig. 1c) [69]. We assumed that the association between all-cause epilepsy and microfilariae prevalence was entirely driven by geographical variation in onchocerciasis prevalence, which we assume to be uncorrelated with other important causes of epilepsy in developing countries, like neurocysticercosis Next, the pre-control number of OAE cases was estimated by multiplying the average OAE prevalence in an area (averaged over pixels) with the size of the population at risk (based on APOC census data), assuming that the population density is homogeneous throughout the area. We stratified the pixels by pre-control nodule prevalence in adult males (> 0%–< 20%, ≥ 20%–< 40%, ≥ 40%) and the population at risk proportional to the number of pixels in each endemicity category. To extrapolate the number of OAE cases to 2015, we assumed that the population at risk and hence the potential number of OAE cases (counterfactual assuming no control) increased annually due to population growth. Population growth between 1995 and 2015 was assumed to be 2.74% based on UN population prospects for SSA [70]. For years that areas remained untreated, we assumed that prevalence of epilepsy remained proportionally stable (i.e. as estimated for 1995). Next, we corrected the number of cases for the presence of MDA, assuming that treatment has no effect on prevalent cases of OAE but prevents incidence of new cases after a scaling-up period of 3 years (i.e. accounting for low treatment coverage in the first few years of MDA programmes). Ivermectin was assumed to reduce OAE incidence to zero (after on three years of non-optimal MDA) on the basis of studies that suggest a reduction in the incidence of epilepsy after ivermectin treatment [26, 27, 71]. We further assumed that once incidence of OAE is zero, the number of prevalent OAE cases declines by 3.5% annually due to mortality, based on a reported 70% cumulative 10-year survival probability among epilepsy cases [16] ($1-\sqrt[10]{0.7}=0.035$). All baseline tables and calculations are shown in Additional file 2. Furthermore, multivariate sensitivity analyses were performed around our assumption of survival probability and number of years of suboptimal ivermectin before OAE incidence drops to zero (Additional file 2).	

In the 18 remaining APOC-areas where OAE was reported or suspected, the total population size in 1995 was 9.2 million people (Table 2). All these 18 areas received treatment with MDA, starting between 1999 and 2012. We predict that the number of OAE cases in those areas was approximately 113 000 (95% *CI*: 53000–371 000), with an overall prevalence of 1.23% of OAE. If we would assume that OAE has a wider geographical distribution among other APOC-areas than those 18 areas, we would expect another 362 000 (95% *CI*: 185000–1 085 000) OAE cases in 1995 (total population size of 81.1 million among all other APOC-areas). We further estimated that approximately 61.5% of all OAE cases were located in onchocerciasis hyperendemic areas (nodule prevalence in adult males ≥40%), 28.7% in mesoendemic areas (20–40% nodule prevalence), and 9.8% in hypoendemic areas (< 20% nodule prevalence).

**Table 2:** Estimated number of onchocerciasis-associated epilepsy cases with 95% confidence intervals in the African Programme for Onchocerciasis Control-areas for two time periods

		1995	2015
Areas where presence of OAE is reported / suspected	Number of cases	93 (*95% CI*: 40–352)	117 (*95% CI*: 50–441)
	Total population	9 214	15 821
Areas where presence of OAE has not yet been investigated	Number of cases	205 (*95% CI*: 85–922)	264 (*95% CI*: 109–1195)
	Total population	81 116	139 282
Total	Number of cases	298 (*95% CI*: 124–1274)	381 (*95% CI*: 158–1636)
	Total population	90 330	155 103

Numbers are presented in thousands

To estimate the number of OAE cases by 2015, we assumed that the number of prevalent cases increased over time due to population growth and that OAE prevalence declined during control of onchocerciasis only due to lower incidence for areas with MDA and excess mortality (i.e. assuming no direct effect of ivermectin on curing epilepsy, hence prevalent OAE cases). We predict that in 2015, there were approximately 117 000 (95% *CI*: 50 000–441 000) prevalent OAE cases, with an overall OAE prevalence of 0.74% (Table 2). If we assume that OAE is also present in onchocerciasis-endemic areas previously under the APOC mandate and where OAE has not (yet) been investigated, we predict an additional 264 000 (95% *CI*: 109 000–1 195 000) cases in 2015.

Of course, there are some limitations in the data and mathematical functions on which this analysis is based. Firstly, the model uses an infection prevalence map [41] based on the Rapid Epidemiological Mapping of Onchocerciasis (REMO) surveys. The REMO surveys have their own inherent challenges, including the use of the less sensitive palpation of nodules as compared to skin snipping. Secondly, the logistic functional relationship for prediction of OAE prevalence by onchocerciasis infection, as reported by Pion et al., includes the at that time available literature for which various corrections needed to be made in order to account for history of treatment and the various diagnostic methods used [4]. These are the best available data to estimate – for now - most accurately the number of OAE cases in APOC-countries. In addition, we applied a more realistic background all-cause epilepsy such as reported by the GBD for SSA (0.36%) rather than the reported background all-cause prevalence epilepsy by Pion et al. (0.17%).

## Quantifying the disease burden: Years of life lived with disability

DALYs are a metric used to quantify the health loss attributable to a disease. They are calculated as the sum of years of life lost (YLLs) due to premature death from a disease and YLDs due to that disease, making DALYs a useful measure for policy purposes because they enable comparison of the importance of diseases. YLDs are calculated by multiplying the number of years lived with a certain disease manifestation with corresponding disability weights. The methods for the calculation of disability weights have been described in detail elsewhere [42, 43].

The GBD study assigned disability weights to more than 300 disorders and diseases, including epilepsy. The disability weight for severe epilepsy is one of the highest with a value of 0.552 (95% *CI*: 0.375–0.710). Other disability weights assigned to epilepsy health states vary in their application by seizure frequency and treatment status. The lowest disability weight is assigned to treatment-controlled, seizure-free epileptics with a value of 0.049 (95% *CI*: 0.031–0.072) (Table 3).

**Table 3:** Different sequela of epilepsy that could be applied to onchocerciasis-associated epilepsy (adapted from [72])

Sequelae	Health State	Lay Description	Disability Weight
Severe epilepsy	Severe (seizures ≥ once per month)	An individual has sudden seizures one or more times each month, with violent muscle contractions and stiffness, loss of consciousness, and loss of urine or bowel control. Between seizures the person has memory loss and difficulty concentrating.	0.552 (0.375–0.71)
Less severe epilepsy	Less severe (seizures < once per month)	An individual has sudden seizures two to five times a year, with violent muscle contractions and stiffness, loss of consciousness, and loss of urine or bowel control.	0.263 (0.173–0.367)
Seizure-free, treated epilepsy	Treated without fits	An individual has a chronic disease that requires medication every day and causes some worry but minimal interference with daily activities.	0.049 (0.031–0.072)

In order to assign disability weights and calculate DALYs attributable to OAE, certain pieces of information are needed:

1. The number of deaths attributable to OAE and the age at death;
2. The frequency of occurrence and severity of seizures (for choosing an applicable health state), and the proportion of patients in each of these health states;
3. The proportion of patients with controlled epilepsy, receiving treatment with any anti-epileptic drug (AED).

Unfortunately, this information is not widely reported in literature. A study in an area of Cameroon highly-endemic for onchocerciasis found that 47% of epilepsy cases in the area experienced at least one seizure in the 6 months prior to the study date while 16% were seizure-free with consistent therapy. At epilepsy onset, 37% had experienced daily seizures [44] (Table 4). DALY calculation for OAE is currently difficult due to the lack of information on the age-distribution of OAE deaths required for calculating YLLs (estimated as the sum difference between age at death and life expectancy at death). However, YLDs can be estimated as the product of the number of prevalent OAE cases and the disability weight for OAE. If the assertions around the epidemiological relationship as published by Pion et al. [4] are representative for the distribution of OAE in all countries previously under the APOC mandate, we estimate that in 2015 there were approximately 39 300 YLDs attributable to OAE in the areas where OAE has been reported or suspected and potentially 88 700 YLDs attributable to OAE in other areas where OAE has not been reported up to now. Calculations can be seen in Table 5.

**Table 4:** Frequency of different health states (indicating different severity levels) of epilepsy in an onchocerciasis hyperendemic area, associated disability weights for each health state (GBD), and calculation of the weighted mean disability weight across health states (weighted for the proportion of cases in each health state, based on Prischich et al. 2008 [44])

Health state	Proportion of epilepsy patients with health state	Disability weight
Severe epilepsy	37%	0.552
Less severe epilepsy	47%	0.263
Seizure-free, treated epilepsy	16%	0.049
Weighted mean disability weight, weighted by the proportion of cases in each health state		0.336

**Table 5:** Methods for calculating YLDs attributable to onchocerciasis-associated epilepsy (OAE)

The disability weight associated with epilepsy depends on the disease severity (see Table 3). We calculated a weighted mean disability weight for epilepsy across the different severity levels, weighting the health state-specific disability weights by the proportion of cases in that health state (Table 4). The proportion of cases in each health state is derived from clinical data of epilepsy severity and frequency in an onchocerciasis hyperendemic area [44]. We assume that the weighted mean disability weights are also applicable to OAE. We multiplied the weighted mean disability weights with the number of prevalent OAE cases to calculate total YLDs attributable to OAE, independently for the various areas. Two types of sensitivity analyses were performed to demonstrate the range in estimates yielded by varying one disability weight value at a time (Additional file 2: Figure S1 and S2). Total YLDs attributable to OAE for 2015 in areas with suspected/reported OAE: 0.336 × 117 000 = *39 300 (95%* *CI: 16800–148 200)* This total estimation of YLDs is based on areas where OAE has been reported or suspected (same 18 areas as stated before). Total YLDs attributable to OAE for 2015 in areas where the presence of OAE has not yet been investigated: 0.336 × 264 000 = *88 700* (*95%* *CI*: *36 600–401 500*) This total estimation of YLDs is based on onchocerciasis-endemic areas previously under the APOC mandate where OAE has not been reported or suspected.	

There are some important limitations to these YLD estimates. First of all, one study is likely not representative of all epilepsy cases in Africa. We have therefore performed an additional sensitivity analysis to assess the robustness of our YLD estimates by comparing our estimated weighted mean disability weight with those of the GBD (Additional file 2: chapter 2). It is likely that the proportion of OAE cases experiencing different levels of epilepsy severity vary by mf intensity level and by treatment history. It is also possible that severity of OAE may vary by geographical location due to different *O. volvulus* species with differing pathogenic potential, such is the case for blindness due to onchocerciasis [45]. Variation is also expected by level of healthcare access, given that a lower disability weight is applied to medically-controlled epilepsy cases. The disability weights from the GBD as shown in Table 3 are not collected for different age groups, and it would be interesting to validate the different assigned severity weights among especially children and young adults with epilepsy in onchocerciasis-endemic areas, as they are the ones with highest OAE prevalence. Ultimately, with so little available published information on the clinical details of the disease, it is hard to know how close this estimate is to the truth. However, the burden of OAE can be substantial as compared to other clinical manifestations of onchocerciasis. If we assume that OAE occurs throughout all onchocerciasis-endemic countries previously under the APOC mandate, the total YLD attributable to OAE would be 128 000 YLDs (39 300 + 88 700 = 128 000 YLDs) in 2015. The GBD estimated 989 653 YLDs due to onchocerciasis (i.e. skin disease, visual impairment, blindness) in the year 2015 for SSA [11]. The actual onchocerciasis burden (in terms of YLDs) would be approximately 12% higher if we would also take account of OAE. Out of the 3.5 million prevalent epilepsy cases in SSA (GBD estimate for 2015 [11]), 11% would be associated with onchocerciasis. Using the weighted mean disability weight for epilepsy, the YLDs due to OAE in APOC-areas forms about 10% of the estimated YLDs in SSA due to epilepsy overall (GBD estimate 2015: 1.31 million YLDs [11]).

## Estimating the socioeconomic burden of OAE

Similar to the distribution of onchocerciasis, OAE occurs almost exclusively in remote areas where people are already disenfranchised by their socioeconomic status. Subsistence farming is generally the primary source of income, and adequate healthcare is often inaccessible [46]. OAE compounds this burden through the accrual of additional direct, indirect and intangible health-related costs [47].

Direct health-related costs include all payable fees related to care-seeking and medical treatment including: payment for transportation to and from a medical facility; costs of diagnostic testing, medication and physician consultation; cost of follow-up consultation and/or hospitalisation; and costs related to home-based care such as the cost accrued from an increased need for personal hygiene products like soap. Beyond the cost of diagnosing and treating OAE, PWE are more likely to acquire other direct health-related costs related to their higher propensity for cooking accidents that may cause severe burns requiring treatment and other incidental injuries. These expenditures reduce the amount of basic financial resources available to the household [48, 49]. Unlike onchocerciasis which has one drug of choice for its control, epilepsy treatments are multiple and their indications are different [50]. Data on the cost of epilepsy management in Africa is currently scarce. Findings from Burundi, Zambia, and South Africa suggest an annual cost of medication alone ranging from US$ 10 to US$ 48 [51–53]. Table 6 shows the average costs of one unit medicine for a PWE (other costs related to the medical management of PWE are currently not available).

**Table 6:** Costs related to medication for treating one person with epilepsy in US$. Adapted from [55]

Name medication	Usage	Median buyer price/day per treated person (US$)^a^	Defined daily dose (DDD)^b^	Median buyer price/year per treated person (US$)	Used by percentage of all epilepsy patients [56]
Phenobarbital ~ 100 mg (1×)	Used for all forms of epilepsy. Most used AED in Sub-Saharan Africa which serves as first-line, because it is relatively cheap and available [10].	$0.0141	100 mg	$5.15	74.6%
Carbamazepine ~ 200 mg (4–5×)	Used for focal seizures [50].	$0.14	1000 mg	$255.50	27.4%
Phenytoin ~ 100 mg (3×)	Used in some generalised seizures and status epilepticus [50].	$0.0449	300 mg	$49.17	22.2%
Valproate ~ 500 mg (3×)	Used for all forms of epilepsy including absences, atonic and myoclonic seizures [50].	$0.1339	1500 mg	$146.62	14.7%
Weighted-average cost of AED^c^					US$ 106.31

Note: ^a^These figures on dosages per drug are based on the daily average dosage that are generally applied in rural African settings, and obtained by comparing several buyer prices for the same product in 2015 [55]

^b^The defined daily dose (DDD) methodology was designed by the WHO to help in following and comparing cost trends at the international level, but not to be used for detailed reimbursement, therapeutic group reference pricing or other specific pricing decisions [55]

^c^The weighted average was calculated by ((100 mg × 1 × cost Phenobarbital unit price × 365 days × 0.746) + (200 mg × 5 × cost Carbamazepine unit price × 365 days × 0.274) + (100 mg × 3 × cost Phenytoin unit price × 365 days × 0.222) + (500 mg × 3 × cost Valproate unit price × 365 days × 0.147))/1.0 total population = US$ 106.31

To estimate the cost of treatment for all OAE cases in APOC countries, we multiplied the predicted number of cases in 2015 by the weighted mean of annual treatment costs of AEDs. No added cost is attributed to account for ivermectin as it is freely distributed by the Mectizan® Donation Programme [54]. We estimate that the total cost for treating all OAE cases in onchocerciasis-endemic areas where OAE has previously been reported or suspected would have been approximately US$12.4 million (117 000 OAE cases × US$ 106.31) in 2015. If OAE would occur in the whole of APOC-areas, we estimate there would be an additional US$28.1 million required (264 000 OAE cases × US$106.31) to treat all additional cases. These figures make up only part of the total direct cost since they do not account for cost of transportation and consultation/hospitalisation. The dosages are currently set to levels that are used in clinical practice of African settings [55]. However, non-adherence of patients to AED may be quite high in some settings (59–63%), overestimating the costs of AEDs as compared to actual usage [9, 56].

Furthermore, these estimates do not reflect the indirect and intangible costs of OAE. Indirect costs are related to lost productivity that is often a consequence of delayed diagnosis and treatment of epilepsy cases due to the limited availability and access to specialists trained in epilepsy care in Africa [57]. Several African countries reported a treatment gap of between 68 and 82% [58–60]. Untreated epilepsy is often associated with lower employment and education levels, and lower socioeconomic status [61]. Children with epilepsy may be banned from school, and adults with epilepsy may be barred from marriage or employment even if seizures do not render their work unsafe [62, 63]. Intangible costs are derived from the emotional and social impact of illness. OAE affects both PWE and caregivers. Caregivers may experience inordinate levels of stress, sleepless nights or burnout related to their responsibility of caring for the patient or their worry about the affected child wandering away [49]. Limited access to AEDs for PWE results in uncontrolled seizures with a high frequency of intellectual disability and psychiatric problems, rendering them extremely vulnerable to abuse and neglect. There have been anecdotal reports that suggest that women with epilepsy in SSA are sexually exploited, abused and have to exchange sex for basic necessities more frequently than unaffected women. This sexual assault also increases their risk for HIV/AIDS and other sexually transmitted infections [62] and if they become pregnant, they may be left with the additional burden of caring for a child [49].

A major challenge in holistically estimating the socioeconomic burden due to OAE is the difficulty in measuring costs because there are many unknown factors (e.g. loss of economy due to time away from work, denial from work of PWE). Therefore, these estimates do not reflect the indirect and intangible costs of OAE. Although we recognise the limitations of providing only costs of medicines, it appears that investing in direct costs (principally treatment with AED) would likely produce benefits in indirect costs (increased productivity) and intangible costs (improved quality of life), all outweighing the initial investment [10, 51]. Such cost estimations assist in making sure necessary resources become available and that the infrastructure can be set in place to target interventions in high-risk onchocerciasis-endemic communities.

## Towards more accurate burden estimates

We have described the major challenges and limitations in our quantification of the number of cases, disease burden estimates (YLDs), and socioeconomic burden. These challenges and limitations can be solved through the acquisition of new and different types of data as well the use of more sophisticated statistical procedures or mathematical models. More data is needed on the prevalence of *O. volvulus* and epilepsy at the community-level of various levels of onchocerciasis endemicity. While some data has already been collected and published [4], there are a number of challenges in utilising it for estimation. Due to the different diagnostic methods and case definitions that are employed in different studies, the measured prevalence cannot be assumed to be comparable due to the divergent sensitivities and specificities. For epilepsy, an adapted case definition applicable in remote areas, including onchocerciasis-endemic areas, to establish aetiology of epilepsy in absence of neuroimaging would help in making study results comparable in future research and comparisons should be made with older diagnostics and case definitions to help equate and interpret results from past research.

Secondly, age- and sex-stratified information is vital in order to capture age- and sex-specific trends in prevalence and disease burden estimates. Epilepsy in onchocerciasis-endemic areas may have a different age pattern in the onset of epilepsy as compared to onchocerciasis non-endemic areas, with a peak onset of epilepsy between ages 10 and 15 years [7, 8, 64]. Age- and sex-stratified data are essential to be able to reproduce disease trends in the prevalence of OAE that can subsequently inform treatment policy, research and drug development efforts targeted at these higher-risk groups. In addition, data on the sex- and age-distribution of OAE deaths is required in order to calculate DALYs. Note that the collection of such data, however, may be quite challenging without the ability to confirm that the epilepsy is caused by onchocerciasis.

Thirdly, there is limited data available about the premature mortality due to epilepsy. In a study in an onchocerciasis-endemic region in Cameroon the relative risk of death among PWE was 6.2 times (95% *CI*: 2.7–14.1) than among those without epilepsy [16]. Additional assessments of excess mortality due to OAE are necessary to refine our assumption of an excess mortality of 3.5% that we applied in the statistical model presented here, based on the study by Kamgno et al. [16]. This would have the effect of a different survival rate of OAE cases (age-stratified), and henceforth a better estimate of the incidence and prevalence of OAE cases across Africa.

Fourthly, very little data is available concerning the current incidence and prevalence of OAE in the majority of sub-Saharan African countries where onchocerciasis is endemic. The available data is concentrated in limited and very focal study sites. This both limits our ability to develop accurate disease burden estimates for vast areas as well as limits our understanding of the epidemiology of the OAE. We have now provided stratified estimates of OAE cases for areas where OAE has been reported or suspected and areas where we do not have any information from. Greater geographical coverage of OAE surveys is essential for making estimates more precise and ensuring that the full burden of OAE is captured.

Lastly, in addition to more refined and robust data, estimates of disease quantification can be refined through the use of modelling frameworks, both statistical and mathematical. Statistical models for the association between infection and morbidity may not well capture non-linearities in population dynamics, but they can make sophisticated estimates of current and future burden. In the past, a Bayesian, hierarchical meta-regression model was used to successfully estimate the burden attributable to epilepsy globally from 1980 to present [32]. Mathematical models may better capture transmission dynamics of onchocerciasis [65–67], such that OAE development is dependent on mf-production with a damage trigger after which epilepsy is allowed to develop. It is possible that damage susceptibility is age-dependent, which could be taken into account in a mathematical model. Likewise, the degree of excess mortality can be accounted for.

## Policy implications

Since epileptic seizures can, under certain circumstances, be well controlled and an individual’s quality of life can be restored with treatment, there are significant gains that can be made for epilepsy patients. The majority of epilepsy patients in Africa do not receive appropriate care, due to limited financial means of households, high costs of AED, lack of proper diagnostics, and/or insufficient number of trained health workers or drug supplies [68]. Scaling-up of care (e.g. additional support and treatment with AED through decentralised services) is urgently needed [10]. The link between onchocerciasis and epilepsy may be exploited in two ways.

Firstly, the possible effect of onchocerciasis control efforts on the incidence of epilepsy may be reason to put in extra resources for the intensification of onchocerciasis elimination activities in highly endemic onchocerciasis areas where high prevalence rates of epilepsy are found [32]. Secondly, health systems can be strengthened in (often remote) highly endemic onchocerciasis areas with high epilepsy prevalence, to enhance timely referral of epilepsy patients (irrespective of the cause of the epilepsy). Community-directed distributors of ivermectin could be trained to identify potential epilepsy cases and refer them to the general health system, to ensure that they receive proper anti-epilepsy treatment. Such efforts may perhaps have little impact on the total epilepsy prevalence in SSA, but it would even so have adjuvant advantages for both onchocerciasis and epilepsy control and may even prevent the potentially significant impact of OAE. In some areas, this may require improvements in accessibility and affordability of healthcare services in order to increase utilisation. Most PWE will respond to AED in stock, at least with a reduction in seizure frequency, and therefore, if they are picked up in the community and referred, will benefit from the health services available.

## Research priorities

We have demonstrated that there is a need to improve estimates of the burden of OAE by country, age and sex, including the calculation of YLLs, YLDs and DALYs attributable to OAE. We have identified six research priorities that need to be addressed in order to improve our understanding of OAE and make our estimates more precise (Table 7). These priorities should be included in the research and policy agendas of both onchocerciasis and epilepsy programmes in Africa. Sustained and intensified funding is required to prompt onchocerciasis elimination efforts in general, with special focus on high transmission zones (often associated with high potential of increased epilepsy prevalence). In addition, these research priorities may motivate health policy-makers to increase funding to health systems across SSA in general, with the aim of tackling epilepsy in these areas.

**Table 7:** Research priorities in the estimation of the current burden of OAE

1	More fundamental research is required to investigate the biological mechanisms of a potential relationship between onchocerciasis and epilepsy. Fundamental evidence of causality could assist in the establishment of burden estimates as well as the potential development of diagnostic algorithm to identify an OAE cases.
2	Repeat the previous performed meta–analysis by Pion et al. [4] including recently performed epilepsy surveys in onchocerciasis-endemic regions to incorporate new information. Sources of bias of included studies should be tracked and a meta-analysis should preferably adjust for potential confounders (age, sex, residence, certain parasitic infections (e.g. NCC)). A correction should be made to exclude epilepsy potentially initiated by other causes.
3	Perform epilepsy incidence or prevalence surveys in onchocerciasis-endemic areas where no data is yet available, using standardised tools for *O. volvulus* and epilepsy diagnosis. Information should be collected on the age and sex distribution of OAE cases (including age of onset of the epilepsy) and the co-prevalence of other sequelae including onchocerciasis associated skin disease (including itching) and ocular disease. Such studies should tempt to include diagnosis of various other parasitic infections, including NCC, malaria, and toxoplasmosis. Muslim or Orthodox Ethiopian-Christian areas where pigs are not raised but endemic for onchocerciasis could be included in such surveys.
4	Design, implement and evaluate a simple tool for ubiquitous use in limited resource settings to identify suspected epilepsy cases, which can be used by community distributors of ivermectin and local primary healthcare workers so that these cases are timely referred to local health facilities.
5	Conduct prospective, longitudinal community intervention trials on the impact of MDA on the incidence of OAE in ivermectin-naïve areas with high onchocerciasis transmission with individual-level follow-up recording *O. volvulus* infection status, epilepsy onset, and ivermectin usage. Compare alternative onchocerciasis control strategies on reducing OAE incidence, e.g. different frequencies of distribution of ivermectin, use of new macrofilaricidal drugs in development, and vector control where feasible.
6	Determine the direct and indirect health-related costs, and intangible costs due to OAE by disease stage, country, sex, and age through a cost-of-illness analysis for a more precise economic burden estimate for OAE.

## Conclusions

Based on our estimates the number of persons with OAE in 2015 is estimated to be 117 000 (95% *CI*: 50 000–441 000) in onchocerciasis-endemic areas where OAE has been reported or suspected and 264 000 (95% *CI*: 109 000–1 195 000) in onchocerciasis-endemic areas where OAE has not yet been investigated. An educated analysis of the burden of OAE is imperative in order to delineate the type and scope of public health responses it requires, both in terms of efficient control interventions and availability of resources. Although the estimates presented here need further refinement, they provide a first step towards quantifying the burden of OAE that we can expect today. These numbers are useful for policy-makers and onchocerciasis and epilepsy programme managers who need to be aware of the public health impact caused by epilepsy in onchocerciasis-endemic areas. Intensification of onchocerciasis control efforts and/or increases in resources for epilepsy healthcare services would then be imperative for most affected areas. People living in onchocerciasis-endemic regions need to understand the full implication and potential gains of supporting and adhering to MDA programmes.

## Additional files

Additional file 1: Multilingual abstracts in the six official working languages of the United Nations. (PDF 654 kb)

Additional file 2: Supplementary methodology, including baseline and summary tables, calculations of OAE cases, and sensitivity analysis. (DOCX 188 kb)

## Abbreviations

95% *CI*: 95% confidence interval
AED: Anti-epileptic drugs
APOC: African Programme for Onchocerciasis Control
DALYs: Disability-adjusted life years
GBD: Global burden of disease
ILAE: International League Against Epilepsy
LF: Lymphatic filariasis
MDA: Mass drug administration
Mf: Microfilariae
NCC: Neurocysticercosis
NP: Nodule prevalence
NTDs: Neglected tropical diseases
OAE: Onchocerciasis-associated epilepsy
*OR*: Odds ratio
PWE: People with epilepsy
PWOE: People without epilepsy
SSA: Sub-Saharan Africa
YLDs: Years of life lived with disability
YLLs: Years of life lost
