# Supplementary material for: Burden of onchocerciasis-associated epilepsy: first estimates and research priorities
Source: Infect Dis Poverty. 2018 Sep 19;7:101. doi: 10.1186/s40249-018-0481-9 (PMC6156959; doi:10.1186/s40249-018-0481-9)
Supplement: Supplementary file 1 — Multilingual abstracts in the six official working languages of the United Nations. (PDF 654 kb) [file 40249_2018_481_MOESM1_ESM.pdf]

Translation of the abstract into the six official working languages of the United Nations

عبء الصرع المصاحب لداء العمى النهري: التقديرات الأولى وأولويات البحث.

ناتالي ف. س. فينكليس ميلتشرز، سارة مولينكوف، روبرت كولبندرز، مايكل إدلينجر، لوك إ. كوفينج، جوليا إيراني، تريسور زولا، جوزيف ن. سيوي، ساكي ج. دي فلاس، أندريا س. وينكلر، ويلما أ. ستولك

الملخص

الخلفية: منذ تسعينات القرن العشرين، تراكمت الأدلة حول انتشارٍ متزايدٍ للصرع في المناطق الموبوءة بداء العمى النهري في أفريقيا مقارنة بالمناطق الخالية من هذا الداء. على الرغم من أن العلاقة السببية بين داء العمى النهري والصرع لم تثبت بعد، فمن المحتمل وجود علاقة بينهما. هنا نناقش الحاجة إلى تقديرات عبء المرض لداء الصرع المرتبط بالعمى النهري (OAE)، وتوفيرها وتفصيل كيفية تنقيح مثل هذه التقديرات ومناقشة تأثيرها الاجتماعي والاقتصادي (OAE)، بما في ذلك تقدير تكلفة العقاقير المضادة للصرع.

المحتوى الرئيسي: قد يساعد تقديم تقديرات عبء مرض الصرع المرتبط بداء العمى النهري OAE في الوقاية من الصرع في المناطق الموبوءة بهذا الداء عن طريق التحفيز على التعاون بين برامج مكافحة داء العمى النهري وخدمات الصحة العقلية. لا يؤثر الصرع بشكل كبير على صحة المصابين فقط، ولكنه يحمل أيضاً عبئًا اجتماعيًا واقتصاديًا كبيرًا على الأسر والمجتمعات المعنية. استخدمنا تقديرات جيولوجية مكانية نُشرت سابقاً عن داء العمى النهري في أفريقيا ونموذج الانحدار اللوجستي المنشور على حدة والذي يحدد الارتباط بين داء العمى النهري والصرع وذلك لتقدير عدد حالات الصرع المرتبط بداء العمى النهري OAE. ثم قمنا بتطبيق قياسات وطأة عجز الصرع لتحديد مقدار العبء من حيث احتساب سنوات الحياة مع العجز (YLD) وتقدير تكلفة العلاج. نقدر أنه في عام 2015 تأثر ما يقرب من 117,000 شخص بمرض الصرع المرتبط بداء العمى النهري في المناطق المتوطنة بهذا الداء، والتي كانت موجودة سابقاً تحت ولاية البرنامج الأفريقي لمكافحة داء العمى النهري (متـ ثيـد (APOCالإبلاغ أو الاشتباه في الصرع المرتبط بداء العمى النهري OAE، و 264,000 شخص آخر في المناطق الموبوءة بمرض العمى النهري حيث لم يتم إجراء أي تحرٍ على الصرع المرتبط بداء العمى النهري من قبل. بلغ العدد الإجمالي لسنوات الحياة مع العجز YLDs بسبب الصرع المرتبط بداء العمى النهري 39,300 و 88,700 في هذه المناطق على التوالي، على أساس قياس متوسط وطأة عجز قدره 0.336. يقرب عبء مرض الصرع المرتبط بالعمى النهري OAE إلى 13 ٪ من مجموع سنوات الحياة مع العجز YLDs التي تعزى إلى مرض العمى النهري و10 ٪ من مجموع سنوات الحياة مع العجز YLDs التي تعزى إلى الصرع. قدرنا أنه بحلول عام 2015، سيكون إجمالي تكاليف العلاج بالعقاقير المضادة للصرع لحالات الصرع المرتبط بالعمى النهري 12.4 مليون دولار أمريكي كحد أدنى.

الاستنتاجات: تشير هذه التقديرات إلى عبء صحي واجتماعي واقتصادي كبير للصرع المرتبط بداء العمى النهري OAE في أفريقيا. إن العلاج والرعاية للأشخاص المصابين بالصرع، وخاصةً في المناطق شديدة التوطن بداء العمى النهري ذي الانتشار الكبير للصرع، يتطلبان بالتالي مزيداً من الموارد المالية والبشرية.

Translated from English version into Arabic by Free bird, proofread by Abdessalam AIT TOUIJAR, through

TRANSLATORS
WITHOUT BORDERS

盘尾丝虫病相关性癫痫疾病负担的首次评估和研究重点

Natalie V.S. Vinkeles Melchers, Sarah Mollenkopf, Robert Colebunders, Michael Edlinger, Luc E. Coffeng, Julia Irani, Trésor Zola, Joseph N. Siewe, Sake J. de Vlas, Andrea S. Winkler, Wilma A. Stolk

摘要
引言：20 世纪 90 年代以来，已有研究表明非洲盘尾丝虫病流行区的癫痫发病率高于非流行区。虽然盘尾丝虫病与癫痫之间的因果关系尚未得到证实，但可能存在关联。本文分析了盘尾丝虫病相关性癫痫(OAE)疾病负担评估的必要性，详细说明应如何改进评估，并讨论了 OAE 的社会经济影响，包括抗癫痫药物的成本估算。
主要内容：OAE 疾病负担的评估能够促进盘尾丝虫病控制计划和精神卫生服务之间的合作，进而推动盘尾丝虫病流行地区癫痫的预防。癫痫病不仅会严重影响患者的健康，还会给相关家庭和社区带来沉重的社会经济负担。通过使用先前发表的关于非洲盘尾丝虫病的地理空间评估和一个单独发表的量化盘尾丝虫病和癫痫相关性的逻辑回归模型，来估算 OAE 病例数。然后应用癫痫残疾权重来量化疾病伤残损失健康生命年（YLD）的负担并估算其治疗费用。评估结果显示，2015 年在盘尾丝虫病流行区约有 11.7 万人受到癫痫的影响，这些区域之前执行的是非洲盘尾丝虫病控制计划(APOC)，该计划曾报道或怀疑 OAE 的发生；另外，生活在盘尾丝虫病流行区的其余 26.4 万人，则未调查过 OAE。加权平均残疾权重为 0.336，这些地区由 OAE 导致的 YLDs 总数分别为 39,300 和 88,700。OAE 的担约占可归因于盘尾丝虫病的 YLD 总数的 13%，占可归因于癫痫的总 YLD 的 10%。至 2015 年，用于 OAE 病例的抗癫痫药物治疗总费用预计至少为 1240 万美元。
结论：上述评估结果表明，非洲国家 OAE 的健康、社会和经济负担相当沉重。因此，对于癫痫患者的治疗和护理，特别是盘尾丝虫病的流行高发区的癫痫患者，需要增加财政和人力资源的投入。

Translated from English version into Chinese by Fan Yang, edited by Pin Yang

CHINESE CENTER FOR DISEASE CONTROL AND PREVENTION
NATIONAL INSTITUTE OF PARASITIC DISEASES

Le fardeau de l'épilepsie due à l'onchocercose : premières estimations et priorités de recherche.

Natalie V.S. Vinkeles Melchers, Sarah Mollenkopf, Robert Colebunders, Michael Edlinger, Luc E. Coffeng, Julia Irani, Trésor Zola, Joseph N. Siewe, Sake J. de Vlas, Andrea S. Winkler, Wilma A. Stolk

Résumé
Contexte: Depuis les années 1990, la recherche met en exergue la prévalence accrue de l'épilepsie dans les régions africaines gangrenées par l'onchocercose comparé à celles qui ne le sont pas. Bien qu'à l'heure actuelle aucun lien de causalité entre l'épilepsie et l'onchocercose n'ait été établi, il existe probablement une relation entre les deux. Nous abordons ici le besoin d'estimations de la charge de morbidité de l'épilepsie due à l'onchocercose (OAE), nous les fournissons, nous détaillons comment ces estimations doivent être perfectionnées et nous traitons la question des conséquences socioéconomiques de l'OAE, notamment l'estimation du coût d'un traitement anti-épileptique.
Discussion: Établir l'estimation de la charge de morbidité de l'OAE pourrait améliorer la prévention de l'épilepsie dans les zones affectées par l'onchocercose en informant et incitant à la coopération entre les programmes de contrôle de l'onchocercose et les services psychiatriques. En plus de ses effets

dévastateurs sur la santé, l'épilepsie est également un lourd fardeau socioéconomique pour les foyers et communautés qu'elle affecte. Afin d'estimer le nombre de personnes atteintes d'OAE, nous avons utilisé d'anciennes estimations géospatiales de l'onchocercose en Afrique ainsi qu'un modèle de regression logistique quantifiant l'association entre l'onchocercose et l'épilepsie. Ensuite, nous avons appliqué les coefficients d'invalidité afin d'estimer ce fardeau en termes de nombre d'années d'incapacité et d'évaluer le coût du traitement. Nous avons estimé qu'en 2015, environ 117 000 personnes souffraient d'OAE dans les régions affectées par l'onchocercose anciennement sous le mandat du Programme Africain de Contrôle de l'onchocercose (APOC) où l'OAE a déjà été signalée ou suspectée, et 264 000 personnes de plus dans les régions affectées par l'onchocercose où l'OAE n'a jamais été étudiée. Le nombre total d'années d'incapacité imputable à l'OAE s'élevait respectivement à 39 300 et 88 700 dans ces régions, basé sur une moyenne pondérée du coefficient d'invalidité de 0,336. Le fardeau de l'OAE est d'approximativement 13% du total du nombre d'années d'incapacité dû à l'onchocercose, et de 10% du total du nombre d'années d'incapacité dû à l'épilepsie. Nous estimons que d'ici 2015, le coût total du traitement de l'épilepsie dans les cas d'OAE s'élèvera au minimum à 12,4 millions de dollars américains.

Conclussions: Ces estimations font état du fardeau sanitaire, social et économique que représente l'OAE en Afrique. En conséquence, le traitement et les soins aux personnes souffrant d'épilepsie, à plus forte raison dans les régions ravagées par l'onchocercose où la prévalence de l'épilepsie est forte, nécessitent de plus amples ressources financières et humaines.

Translated from English version into French by William Squire, proofread by Eve Anderson, through

TRANSLATORS
WITHOUT BORDERS

Бремя эпилепсии, связанной с онхоцеркозом: предварительная оценка и приоритеты исследований.

Натали В.С. Винкелес Мельхерс, Сара Молленкопф, Роберт Колебундерс, Михаэль Эдлингер, Люк Э. Коффенг, Юлия Ирани, Трезор Золя, Жозеф Н. Сью, Саке Ж. де Влас, Андреа С. Винклер, Вильма А. Столк

Аннотация

Общие сведения: С 1990-х годов всё больше свидетельств повышения распространенности эпилепсии в районах Африки с высоким уровнем заболеваемости онхоцеркозом (в отличие от районов, где онхоцеркоз не распространен). Хотя наличие причинно-следственной связи между онхоцеркозом и эпилепсией пока не доказано, некоторая связь между ними, вероятно, существует. В настоящей статье рассматривается, почему необходимо оценить бремя эпилепсии, связанной с онхоцеркозом (ЭСО), представлены результаты такой оценки, описано, как можно уточнить эти результаты, и рассмотрены социально-экономические последствия ЭСО, включая оценку расходов на противоэпилептические препараты.

Основная часть: Проведение оценки бремени ЭСО может способствовать предотвращению распространения эпилепсии в районах с высоким уровнем заболеваемости онхоцеркозом, в том числе путем налаживания сотрудничества между программами по борьбе с онхоцеркозом и психиатрическими службами. Эпилепсия не только оказывает существенное влияние на здоровье больных, но и является большим социально-экономическим бременем для связанных с ними домохозяйств и общин. Мы использовали ранее опубликованные геопространственные данные по распространению онхоцеркоза в Африке и отдельно опубликованную логистическую регрессионную модель, количественно выражающую связь между онхоцеркозом и эпилепсией, чтобы оценить количество случаев ЭСО. Затем мы применили весовой коэффициент инвалидности для эпилепсии для количественного выражения бремени в виде количества лет, прожитых с инвалидностью (ЛПИ), и оценили расходы на лечение. По нашим оценкам в 2015 году было около 117 000 человек с ЭСО в районах с высоким уровнем заболеваемости онхоцеркозом, которые ранее находились под контролем Африканской программы по борьбе с онхоцеркозом и где были сообщения и подозрения на случаи ЭСО, и еще 264 000 человек в районах, где онхоцеркоз распространен, но случаи ЭСО ранее исследованы не были. Общее количество ЛПИ по причине ЭСО для этих районах составило 39 300 и 88 700, соответственно, а средневзвешенная величина весового коэффициента инвалидности составила 0,336. Бремя ЭСО составляет примерно 13% от общего количества ЛПИ, которые можно отнести на счет онхоцеркоза, и 10% от общего количества ЛПИ, которые можно отнести на счет эпилепсии. По нашим оценкам к 2015 году общая сумма расходов на противоэпилептические препараты для лечения ЭСО составит минимум 12,4 миллионов долларов США.
Выводы: Проведенная оценка позволяет сделать предположение о значительном бремени ЭСО в Африке с точки зрения здравоохранения и с социально-экономической точки зрения. Таким образом, для лечения и ухода за людьми с эпилепсией, особенно в районах с высоким уровнем заболеваемости онхоцеркозом и эпилепсией, требуется больше финансовых и человеческих ресурсы.

Translated from English version into Russian by Daria, proofread by Alexander Somin, through

TRANSLATORS
WITHOUT BORDERS

Carga de la epilepsia asociada a la oncocercosis: primeras estimaciones y prioridades del estudio

Natalie V.S. Vinkeles Melchers, Sarah Mollenkopf, Robert Colebunders, Michael Edlinger, Luc E. Coffeng, Julia Irani, Trésor Zola, Joseph N. Siewe, Sake J. de Vlas, Andrea S. Winkler, Wilma A. Stolk

Resumen

Contexto: Desde los años 90, se han acumulado pruebas de una mayor prevalencia de la epilepsia en las áreas endémicas de la oncocercosis en África en comparación con las áreas libres de oncocercosis.

Aunque aún debe comprobarse la relación causal entre la oncocercosis y la epilepsia, es probable que estén vinculadas. En este documento se discute la necesidad de realizar estimaciones de la carga de morbilidad de la epilepsia asociada a la oncocercosis (OAE, por sus siglas en inglés), se proporcionan dichas estimaciones, se detalla cómo estas deberían refinarse y se explica el impacto socioeconómico de la OAE, incluyendo una estimación del coste de los medicamentos antiepilépticos.

Cuerpo principal: Proporcionar las estimaciones de la carga de la OAE puede ayudar a prevenir la epilepsia en las áreas endémicas de oncocercosis incitando a la colaboración entre los programas de control de la oncocercosis y los servicios de salud mental e informando sobre ella. La epilepsia no solo tiene un impacto muy negativo en aquellas personas afectadas, sino que también implica una importante carga socioeconómica para las viviendas y las comunidades involucradas. Se han utilizado estimaciones geoespaciales de la oncocercosis en África publicadas con anterioridad y un modelo de regresión logística publicado por separado que cuantifica el vínculo entre la oncocercosis y la epilepsia para estimar el número de casos de OAE. Luego se aplicaron cargas de discapacidad a la epilepsia para cuantificar la carga en relación con los años vividos con una discapacidad (AVD) y estimar el coste del tratamiento. Se estima que en 2015, apenas 117 000 personas se vieron afectadas por la OAE por todas las áreas endémicas de la oncocercosis que anteriormente habían estado acogidas en el Programa Africano de Control de la Oncocercosis (APOC, por sus siglas en inglés) en las que se ha sospechado o informado de casos de OAE y otras 264 000 personas en áreas endémicas de la oncocercosis donde nunca se ha investigado la OAE. El número total de AVD debido a la OAE era de 39 000 y 87 000 en estas áreas respectivamente, en relación a una carga de discapacidad media de 0,336. La carga de la OAE es de aproximadamente el 13 % del total de AVD atribuibles a la oncocercosis y el 10 % del total de AVD atribuibles a la epilepsia. Se estima que para 2015 el coste total del tratamiento con medicamentos antiepilépticos para los casos de OAE habría sido de un mínimo de 12,4 millones de dólares estadounidenses.

Conclusiones: Estas estimaciones sugieren una carga sanitaria, social y económica considerable de la OAE en África. El tratamiento y los cuidados para las personas con epilepsia, especialmente en áreas hiperendémicas de oncocercosis con alto predominio de la epilepsia, requieren, por tanto, más recursos económicos y humanos.

Translated from English version into Spanish by Ribcamar, proofread by Felipe Chávez, through

TRANSLATORS
WITHOUT BORDERS
