# Supplementary material for: Burden of onchocerciasis-associated epilepsy: first estimates and research priorities
Source: Infect Dis Poverty. 2018 Sep 19;7:101. doi: 10.1186/s40249-018-0481-9 (PMC6156959; doi:10.1186/s40249-018-0481-9)
Supplement: Supplementary file 2 — Supplementary methodology, including baseline and summary tables, calculations of OAE cases, and sensitivity analysis. (DOCX 188 kb) [file 40249_2018_481_MOESM2_ESM.docx]

**Burden of onchocerciasis-associated epilepsy: first estimates and research priorities - Supplement**

Natalie V.S. Vinkeles Melchers^1*^, Sarah Mollenkopf^2^, Robert Colebunders^3^, Michael Edlinger^4^, Luc E. Coffeng^1^, Julia Irani^5^, Trésor Zola^6^, Joseph N. Siewe^7^, Sake J. de Vlas^1^, Andrea S. Winkler^8^, Wilma A. Stolk^1^

^1^ Department of Public Health, Erasmus MC, University Medical Center Rotterdam, P.O. box 2040, 3000 CA Rotterdam, The Netherlands;

Email: [N.vinkelesmelchers@erasmusmc.nl](mailto:N.vinkelesmelchers@erasmusmc.nl) (MSc. MPH.)

^2^ Institute for Health Metrics and Evaluation, University of Washington, 2301 5^th^ Avenue, Suite 600, Seattle, WA 98121, USA

Email: [smollenk@uw.edu](mailto:smollenk@uw.edu) (MPH.)

^3^ Global Health Institute, University of Antwerp, Antwerp, Belgium; Department of Clinical Sciences, Institute of Tropical Medicine, Antwerp, Belgium

Email: [robert.colebunders@uantwerpen.be](mailto:robert.colebunders@uantwerpen.be) (Prof. MD.)

^4^ Department of Medical Statistics, Informatics, and Health Economics, Medical University Innsbruck, Austria

Email: [Michael.Edlinger@i-med.ac.at](mailto:Michael.Edlinger@i-med.ac.at) (PhD. MSc.)

^1^ Department of Public Health, Erasmus MC, University Medical Center Rotterdam, P.O. box 2040, 3000 CA Rotterdam, The Netherlands;

Email: [l.coffeng@erasmusmc.nl](mailto:l.coffeng@erasmusmc.nl) (PhD. MD.)

^5^ Department of Public Health, Institute of Tropical Medicine Antwerp, Nationalestraat 155, 2000, Antwerp, Belgium

Email: [jirani@itg.be](mailto:jirani@itg.be) (MPhil, MSc.)

^6^ University of Kinshasa, Department of Tropical Medicine, Democratic Republic of the Congo

Email: [zolanga@yahoo.fr](mailto:zolanga@yahoo.fr) (MD.)

^7^ Global Health Institute, University of Antwerp, Antwerp, Belgium

Email: [josephnelson.siewefodjo@uantwerpen.be](mailto:josephnelson.siewefodjo@uantwerpen.be) (MD.)

^1^ Department of Public Health, Erasmus MC, University Medical Center Rotterdam, P.O. box 2040, 3000 CA Rotterdam, The Netherlands;

Email: [s.devlas@erasmusmc.nl](mailto:s.devlas@erasmusmc.nl) (Prof. PhD.)

^8^ Centre for Global Health, Institute for Health and Society, Oslo, Norway and Center for Global Health, Department of Neurology, Technical University of Munich, Germany;

Email: [a.s.winkler@medisin.uio.no](mailto:a.s.winkler@medisin.uio.no) (Prof. MD.)

^1^ Department of Public Health, Erasmus MC, University Medical Center Rotterdam, P.O. box 2040, 3000 CA Rotterdam, The Netherlands;

Email: [w.stolk@erasmusmc.nl](mailto:w.stolk@erasmusmc.nl) (PhD.)

*Correspondence to:

Natalie V.S. Vinkeles Melchers, Department of Public Health, Erasmus MC, University Medical Center Rotterdam, P.O. box 2040, 3000 CA Rotterdam, The Netherlands; [N.vinkelesmelchers@erasmusmc.nl](mailto:N.vinkelesmelchers@erasmusmc.nl); Natalie.melchers@gmail.com; +31 (0)10 70 38465

**Table S1. Characteristics of each African Programme for Onchocerciasis Control (APOC)-project (country, project name, population for 1995 (pre-control), start year of community-directed treatment with ivermectin (MDA), and whether OAE has been reported or suspected in the area) and estimated pre-control (1995) number of OAE cases .**

| **Country** | **Project** | **Population project**  **1995^1^** | **MDA start**  **year^2^** | **Presence of OAE reported or suspected (i.e. reported in literature)** | **Pre-control OAE cases^3^** | | |
| --- | --- | --- | --- | --- | --- | --- | --- |
|  |  |  |  |  | **Lower bound (95%CI)** | **Point estimate** | **Upper bound (95%CI)** |
| Angola | Bengo | 15,131 | 2010 | No | 0 | 0 | 38 |
| Angola | Benguela | 28,942 | 2012 | No | 0 | 0 | 97 |
| Angola | Cuanza Norte | 14,790 | 2011 | No | 0 | 0 | 50 |
| Angola | Huila | 136,181 | 2010 | No | 127 | 337 | 1,583 |
| Angola | Kuando Kubango | 234,779 | 2009 | No | 0 | 0 | 487 |
| Angola | Lunda Norte | 175,409 | 2009 | No | 13 | 111 | 979 |
| Angola | Lunda sul | 147,786 | 2009 | No | 0 | 68 | 771 |
| Angola | Moxico 1 | 154,449 | 2011 | No | 0 | 0 | 382 |
| Angola | Namibe | 20,423 | >=2015 | No | 0 | 0 | 75 |
| Angola | NY Benguela | 65,013 | 2014 | No | 0 | 23 | 321 |
| Angola | NY Cuanza Norte | 10,607 | 2014 | No | 0 | 0 | 15 |
| Angola | NY Huila | 13,162 | 2014 | No | 0 | 5 | 64 |
| Angola | NY Lunda Norte | 38,214 | 2014 | No | 0 | 0 | 117 |
| Angola | NY Moxico 1 | 197,151 | 2014 | No | 0 | 0 | 326 |
| Angola | P5Angola | 149,986 | >=2015 | No | 0 | 0 | 82 |
| Angola | Uige | 108,412 | 2014 | No | 23 | 95 | 673 |
| Angola | Zaire | 8,177 | 2014 | No | 0 | 0 | 15 |
| Burundi | Bururi | 252,262 | 2008 | Yes | 9 | 118 | 1,216 |
| Burundi | Cibitoke-Bubanza | 602,375 | 2006 | No | 489 | 1,270 | 6,084 |
| Burundi | P5Burundi | 475,130 | >=2015 | No | 0 | 0 | 510 |
| Burundi | Rutana | 196,386 | 2008 | No | 0 | 0 | 426 |
| Cameroon | Adamaoua 1 | 328,738 | 2008 | No | 303 | 807 | 3,812 |
| Cameroon | Adamaoua 2 | 298,708 | 2004 | No | 341 | 863 | 3,857 |
| Cameroon | Centre 1 | 303,593 | 2003 | Yes | 1,330 | 3,041 | 11,225 |
| Cameroon | Centre 2 | 71,459 | 2005 | No | 202 | 479 | 1,866 |
| Cameroon | Centre 3 | 230,735 | 2004 | Yes | 478 | 1,125 | 4,484 |
| Cameroon | East | 84,274 | 2007 | No | 195 | 473 | 1,902 |
| Cameroon | Far North | 199,321 | 2007 | No | 0 | 0 | 314 |
| Cameroon | Littoral 1 | 199,274 | 2007 | Yes | 905 | 2,069 | 7,621 |
| Cameroon | Littoral 2 | 106,306 | 2003 | Yes | 711 | 1,659 | 6,094 |
| Cameroon | Northern | 446,950 | 2004 | No | 1,272 | 2,975 | 11,475 |
| Cameroon | Northwest | 580,659 | 2005 | No | 1,584 | 3,566 | 13,426 |
| Cameroon | P20Cameroon | 106,977 | >=2015 | No | 277 | 627 | 2,379 |
| Cameroon | P5Cameroon | 1,064,406 | >=2015 | No | 0 | 0 | 3,240 |
| Cameroon | South | 212,215 | 2006 | No | 182 | 473 | 2,239 |
| Cameroon | South West 1 | 278,230 | 2005 | No | 1,598 | 3,658 | 13,348 |
| Cameroon | South West 2 | 189,350 | 2004 | No | 848 | 1,919 | 7,020 |
| Cameroon | Western | 1,163,347 | 2003 | Yes | 9,108 | 21,167 | 77,238 |
| CAR | CAR region 3 | 303,529 | 2003 | Yes | 1,568 | 3,541 | 12,833 |
| CAR | CAR region 4 | 316,242 | 2003 | No | 586 | 1,389 | 5,627 |
| CAR | CAR region 5 | 292,541 | 2003 | No | 162 | 504 | 2,735 |
| CAR | CAR region 6 | 450,128 | 2003 | No | 1,480 | 3,404 | 12,837 |
| CAR | P20CAR | 44,318 | >=2015 | No | 96 | 219 | 850 |
| CAR | P5CAR | 100,665 | >=2015 | No | 0 | 32 | 487 |
| Chad | Chad | 1,222,831 | 2001 | No | 1,067 | 2,711 | 12,717 |
| Chad | P5 Chad propext | 87,533 | >=2015 | No | 0 | 0 | 100 |
| Chad | P5Chad | 152,159 | >=2015 | No | 42 | 153 | 1,011 |
| Congo | Congo 1 | 557,645 | 2007 | No | 183 | 638 | 4,001 |
| Congo | P20Congo | 24,361 | 2014 | No | 118 | 263 | 948 |
| Congo | P5Congo | 327,184 | >=2015 | No | 0 | 0 | 1,213 |
| DRC | Bandundu | 3,886 | 2005 | No | 0 | 0 | 14 |
| DRC | Bas-Congo Kinshasa | 920,210 | 2008 | No | 1,516 | 3,586 | 14,708 |
| DRC | Butembo-Beni | 571,782 | 2011 | No | 1,498 | 3,378 | 12,787 |
| DRC | Equateur-Kiri | 759,902 | 2009 | No | 1,672 | 3,795 | 14,672 |
| DRC | Ituri-Nord | 768,903 | 2009 | Yes | 5,996 | 14,177 | 52,292 |
| DRC | Ituri-Sud | 702,859 | 2012 | Yes | 4,311 | 10,144 | 37,646 |
| DRC | Kasai | 6,592,571 | 2009 | No | 14,177 | 35,267 | 144,559 |
| DRC | Kasongo | 825,854 | 2009 | No | 599 | 1,749 | 8,878 |
| DRC | Katanga-Nord | 383,688 | 2009 | No | 772 | 1,901 | 7,807 |
| DRC | Katanga-Sud | 424,988 | 2009 | Yes | 848 | 2,108 | 8,704 |
| DRC | Lualaba | 137,852 | 2008 | No | 309 | 708 | 2,756 |
| DRC | Lubutu | 204,773 | 2009 | No | 1,009 | 2,263 | 8,173 |
| DRC | Masisi-Walikale | 641,800 | 2010 | No | 2,793 | 6,402 | 23,656 |
| DRC | Mongala | 892,449 | 2009 | No | 1,534 | 3,576 | 14,466 |
| DRC | NY Katanga-Nord | 278,410 | 2014 | No | 0 | 0 | 459 |
| DRC | NY Lualaba | 602,521 | 2014 | No | 0 | 28 | 2,533 |
| DRC | NY Masisi-Walikale | 33,446 | 2014 | No | 38 | 94 | 413 |
| DRC | NY Rutshuru-Ngoma | 5,206 | 2014 | No | 18 | 39 | 142 |
| DRC | NY Sankuru | 287,456 | 2014 | No | 69 | 311 | 2,122 |
| DRC | NY Ueles | 97,792 | 2014 | Yes | 632 | 1,475 | 5,437 |
| DRC | P20DRC | 1,441,647 | >=2015 | No | 4,980 | 11,371 | 42,502 |
| DRC | P5DRC | 4,584,358 | >=2015 | No | 0 | 0 | 14,571 |
| DRC | Rutshuru-Ngoma | 404,229 | 2009 | No | 583 | 1,327 | 5,418 |
| DRC | Sankuru | 653,966 | 2007 | No | 2,946 | 7,133 | 27,328 |
| DRC | Tshopo | 976,905 | 2010 | Yes | 4,342 | 10,028 | 37,243 |
| DRC | Tshuapa | 870,370 | 2010 | No | 3,514 | 8,066 | 29,986 |
| DRC | Ubangi-Nord | 490,091 | 2011 | No | 2,057 | 4,797 | 17,991 |
| DRC | Ubangi-Sud | 826,671 | 2011 | No | 720 | 1,831 | 8,598 |
| DRC | Ueles | 964,070 | 2006 | Yes | 7,018 | 16,578 | 61,216 |
| Equatorial Guinea | P5EqGuinea | 159,367 | >=2015 | No | 0 | 0 | 0 |
| Equatorial Guinea | Bioko | 50,364 | 2007 | No | 0 | 0 | 0 |
| Ethiopia | Assosa | 363,499 | 2014 | No | 61 | 284 | 2,151 |
| Ethiopia | Bench-Maji | 480,637 | 2005 | No | 939 | 2,109 | 8,201 |
| Ethiopia | East Wellega | 588,599 | 2006 | No | 718 | 1,717 | 7,398 |
| Ethiopia | Gambella | 70,322 | 2006 | No | 107 | 249 | 1,025 |
| Ethiopia | Horo Guduru | 34,514 | 2014 | No | 42 | 97 | 411 |
| Ethiopia | Illubabor | 497,845 | 2004 | No | 1,047 | 2,371 | 9,194 |
| Ethiopia | Jimma | 587,663 | 2004 | No | 175 | 612 | 3,950 |
| Ethiopia | Kaffa-Sheka | 834,630 | 2003 | No | 2,106 | 4,811 | 18,426 |
| Ethiopia | Kamashi | 310,379 | 2014 | No | 635 | 1,465 | 5,777 |
| Ethiopia | Metekel | 106,226 | 2007 | No | 60 | 167 | 889 |
| Ethiopia | North Gondar | 206,260 | 2004 | No | 32 | 181 | 1,362 |
| Ethiopia | NY East Wellega | 188,407 | 2014 | No | 390 | 881 | 3,422 |
| Ethiopia | NY West Wellega | 191,358 | 2014 | No | 54 | 182 | 1,192 |
| Ethiopia | P20Ethiopia | 218,854 | 2014 | No | 540 | 1,210 | 4,585 |
| Ethiopia | P5Ethiopia | 2,310,931 | 2014 | No | 0 | 264 | 9,250 |
| Ethiopia | West Shewa | 38,047 | 2014 | No | 0 | 0 | 62 |
| Ethiopia | West Wellega | 675,881 | 2006 | No | 1,717 | 3,950 | 15,215 |
| Gabon | P5Gabon | 56,678 | >=2015 | No | 0 | 0 | 69 |
| Malawi | Malawi Extension | 761,595 | 2004 | No | 0 | 0 | 1,994 |
| Malawi | Thyolo Mwanza | 580,860 | 2004 | No | 346 | 898 | 4,619 |
| Mozambique | P20Mozambique | 10,596 | >=2015 | No | 0 | 1 | 38 |
| Mozambique | P5Mozambique | 30,951 | >=2015 | No | 0 | 0 | 27 |
| Nigeria | Adamawa | 1,166,497 | 2001 | No | 0 | 0 | 1,707 |
| Nigeria | Akwa Ibom | 19,971 | 2006 | No | 0 | 0 | 47 |
| Nigeria | Bauchi | 1,195,154 | 2009 | No | 0 | 0 | 930 |
| Nigeria | Benue | 2,357,066 | 2007 | No | 4,285 | 9,924 | 39,707 |
| Nigeria | Borno | 939,540 | 2006 | No | 0 | 0 | 2,694 |
| Nigeria | Cross River | 851,884 | 1999 | No | 646 | 1,788 | 8,881 |
| Nigeria | Edo Delta | 1,095,229 | 1999 | No | 425 | 1,326 | 7,881 |
| Nigeria | Ekiti | 1,478,542 | 2004 | No | 0 | 386 | 6,091 |
| Nigeria | Enugu Anambra Ebony | 1,627,437 | 1999 | No | 5,890 | 13,258 | 48,892 |
| Nigeria | FCT | 345,498 | 2004 | No | 0 | 0 | 106 |
| Nigeria | Gombe | 1,298,651 | 2006 | No | 0 | 0 | 3,782 |
| Nigeria | Imo Abia | 897,152 | 1999 | Yes | 657 | 1,734 | 8,556 |
| Nigeria | Jigawa | 243,207 | 2004 | No | 0 | 0 | 0 |
| Nigeria | Kaduna | 1,889,678 | 1992 | No | 0 | 581 | 9,637 |
| Nigeria | Kano | 674,286 | 2000 | No | 0 | 0 | 1,116 |
| Nigeria | Kebbi | 141,685 | 2006 | No | 0 | 0 | 0 |
| Nigeria | Kogi | 1,217,463 | 1999 | No | 220 | 968 | 7,221 |
| Nigeria | Kwara | 1,028,996 | 2000 | No | 444 | 1,274 | 7,291 |
| Nigeria | Niger | 1,808,862 | 2004 | No | 0 | 0 | 6,212 |
| Nigeria | Ogun | 234,347 | 2003 | No | 138 | 360 | 1,865 |
| Nigeria | Ondo | 932,165 | 2001 | No | 421 | 1,166 | 6,558 |
| Nigeria | Osun | 1,099,749 | 2009 | No | 489 | 1,343 | 7,579 |
| Nigeria | Oyo | 750,013 | 2011 | No | 258 | 783 | 4,822 |
| Nigeria | P20Nigeria | 151,484 | 2014 | No | 326 | 727 | 2,779 |
| Nigeria | P5Nigeria | 5,143,695 | >=2015 | No | 0 | 0 | 5,621 |
| Nigeria | Plateau Nassarawa | 1,042,881 | 2000 | No | 0 | 0 | 2,249 |
| Nigeria | Plateau Nassarawa LF | 1,055,859 | 2000 | No | 911 | 2,345 | 11,073 |
| Nigeria | Taraba | 1,134,636 | 2009 | No | 1,022 | 2,773 | 13,244 |
| Nigeria | Yobe | 419,646 | 2002 | No | 0 | 0 | 624 |
| Nigeria | Zamfara | 194,755 | 1999 | No | 0 | 0 | 0 |
| South Sudan | East Bahr El Ghazal | 373,631 | 2011 | No | 539 | 1,242 | 5,112 |
| South Sudan | East Equatoria | 664,116 | 2009 | No | 767 | 1,851 | 8,080 |
| South Sudan | P20SouthSudan | 18,709 | >=2015 | No | 36 | 81 | 316 |
| South Sudan | P5SouthSudan | 514,979 | >=2015 | No | 0 | 0 | 725 |
| South Sudan | Upper Nile | 348,000 | 2010 | No | 0 | 1 | 1,343 |
| South Sudan | West Bahr El Ghazal | 2,013,895 | 2011 | No | 2,028 | 5,371 | 24,938 |
| South Sudan | West Equatoria | 475,026 | 2009 | Yes | 630 | 1,513 | 6,445 |
| Sudan | P5Sudan | 145,016 | >=2015 | No | 0 | 0 | 74 |
| Sudan | Sudan | 136,979 | 2008 | No | 0 | 0 | 0 |
| Sudan | Sudan Abu Hamed | 136,979 | 2008 | No | 0 | 0 | 0 |
| Tanzania | Kilosa | 328,588 | 2004 | No | 0 | 0 | 972 |
| Tanzania | Mahenge | 328,510 | 2003 | Yes | 974 | 2,235 | 8,474 |
| Tanzania | Morogoro | 235,141 | 2006 | No | 79 | 266 | 1,650 |
| Tanzania | P5Tanzania | 595,822 | >=2015 | No | 0 | 0 | 1,876 |
| Tanzania | Ruvuma | 259,587 | 2002 | No | 1,476 | 3,423 | 12,597 |
| Tanzania | Tanga | 203,653 | 2004 | No | 0 | 0 | 615 |
| Tanzania | Tukuyu | 74,528 | 2001 | No | 152 | 359 | 1,435 |
| Tanzania | Tunduru | 80,815 | 2005 | No | 265 | 599 | 2,232 |
| Uganda | P5Uganda | 149,877 | 1997 | No | 0 | 0 | 412 |
| Uganda | Phase 1 | 234,781 | 2001 | Yes | 0 | 33 | 1,078 |
| Uganda | Phase 2 | 456,807 | 2000 | No | 0 | 0 | 0 |
| Uganda | Phase 3 | 842,581 | 2003 | No | 2,907 | 6,931 | 26,701 |
| Uganda | Phase 4 | 476,936 | 1999 | Yes | 0 | 157 | 2,323 |
| Uganda | Phase 5 | 307,101 | 2012 | Yes | 43 | 299 | 2,251 |
| **TOTAL** |  | **90,329,648** |  |  | **124,241** | **298,454** | **1,274,357** |

^1^ Source: APOC treatment database, based on the APOC census conducted by community drug distributors for estimating the amount of ivermectin required in mass treatments.

^2^ Ongoing projects (as of November 2013): The first year with treatment coverage greater than 60% was used, as effective control of the disease requires the treatment coverage of 60% and above. New projects: The start year was predicted based on APOC’s strategic plan to focus on the onchocerciasis elimination for the next decade 2016-2025, the current epidemiology, and the current political situation. All projects were assumed to have a treatment frequency per annum.

^3^ Number of pre-control OAE cases are based on pixel-level estimates of the prevalence of OAE using a functional relationship as published by Pion *et al.*  [1], given the pixel-level prevalence of mf,.

**Table S2. Detailed summary table of OAE cases per MDA start year and by areas where presence of OAE has been reported or suspected.**

| **MDA start**  **year** | **Areas where presence of OAE is reported / suspected** | | | **Areas where presence of OAE has not yet been investigated** | | |
| --- | --- | --- | --- | --- | --- | --- |
|  | **No. of OAE cases (1995)** | **No. of OAE cases three years after start of MDA^1^** | **No. of OAE cases (2015)^2^** | **No. of OAE cases (1995)** | **No. of OAE cases three years after start of MDA^1^** | **No. of OAE cases (2015)^2^** |
| 1997 | - | - | - | - | - | - |
| 1999 | 1,891 | 2,285 | 1,437 | 17,340 | 20,952 | 13,178 |
| 2000 | - | - | - | 3,618 | 4,492 | 2,928 |
| 2001 | 33 | 42 | 28 | 4,236 | 5,402 | 3,649 |
| 2002 | - | - | - | 3,423 | 4,486 | 3,140 |
| 2003 | 31,642 | 42,599 | 30,902 | 17,399 | 23,424 | 16,992 |
| 2004 | 1,125 | 1,557 | 1,170 | 10,203 | 14,113 | 10,609 |
| 2005 | - | - | - | 10,411 | 14,795 | 11,526 |
| 2006 | 16,578 | 24,203 | 19,540 | 7,924 | 11,569 | 9,340 |
| 2007 | 2,069 | 3,103 | 2,596 | 18,335 | 27,503 | 23,010 |
| 2008 | 118 | 182 | 158 | 5,101 | 7,861 | 6,815 |
| 2009 | 17,798 | 28,180 | 25,321 | 56,025 | 88,706 | 79,704 |
| 2010 | 10,028 | 16,313 | 15,190 | 15,387 | 25,030 | 23,307 |
| 2011 | - | - | - | 17,402 | 29,083 | 28,064 |
| ≥2012 | 11,919 | 20,465 | 20,465 | 18,449 | 31,678 | 31,678 |
| **TOTAL** | **93,201** |  | **116,808** | **205,253** |  | **263,943** |

^1^ From 1995 onwards, the pre-control number of OAE cases is assumed to increase due to population growth (annual increase of 2.74%) until three years after start of MDA, at which point we assume incidence is zero due to successful implementation of MDA. For projects that started MDA in 2012 or later, this column reports the estimated number of OAE cases in 2015.

^2^ The number of OAE cases in 2015 was calculated based on the estimated number of cases three years after start of MDA, assuming that no new cases occur from then onwards and that the number of prevalent cases decreases by 3.5% each year due to mortality, based on a reported 70% cumulative 10-year survival probability among epilepsy cases in sub-Sahara Africa [2].

# Sensitivity analysis

## *1. User-defined disease parameters in estimating number of OAE cases in 2015*

We have performed a sensitivity analysis around the assumption of the annual cumulative survival probability of OAE cases. The baseline factor for survival was set at 0.96 ($\sqrt[10]{0.7}=0.035$), based on cohort study results from Kamgno *et al.* [2]. We have assessed the impact of a worsened annual survival probability of OAE cases by applying a factor of 0.85 (see **Table S3**). This was an arbitrarily chosen cut-off (with presumably a high rate of mortality), as no other evidence is available from rural Africa. On the contrary, standardised mortality ratios (SMR) from industrialised countries were between SMR 2.1 and 3.6 [3–5], with all-cause mortality due to epilepsy up to a SMR of 7.6 in individuals <20 years of age [3]. Another study looking at the cumulative survival of, among others, idiopathic epilepsy patients found a survival of ~45% of patients after 10 years follow-up time (1 – ($\sqrt[10]{0.45}=0.077)=0.923$) [6]. We have added this number to our sensitivity analysis.

Furthermore, we assume that it took an average APOC project approximately three years to provide MDA with optimal population coverage (>60%). Without optimal treatment coverage, the effect of ivermectin on the incidence of epilepsy was considered nil. However, the duration to reach optimal population coverage with ivermectin treatment may vary per APOC project, and the average duration may be lower or higher. We have therefore assessed the impact of the number of years required for APOC-projects to reach optimal population coverage with ivermectin (range: 1 – 5 years) .

**Table S3. Results of the sensitivity analysis around the assumptions in the model that may impact the estimated number of OAE cases in SSA by 2015. Number of cases reported in thousands [95%CI].**

|  | | **Areas where presence of OAE is reported or suspected** | **Areas where presence of OAE has not yet been investigated** |
| --- | --- | --- | --- |
| **Assumed annual cumulative survival probability of OAE cases** | | | |
|  | **0.965 (baseline)** | 117 [50 - 441] | 264 [109 - 1,195] |
|  | **0.923** | 96 [41 - 362] | 222 [91 – 1,010] |
|  | **0.850** | 71 [30 - 267] | 169 [70 - 781] |
| **Number of years of MDA required before OAE incidence drops to zero** | | | |
|  | **1 year** | 103 [44 – 390] | 237 [98 – 1,077] |
|  | **3 years (baseline)** | 117 [50 - 441] | 264 [109 - 1,195] |
|  | **5 years** | 130 [55 - 489] | 293 [121 – 1,321] |

## *2. Sensitivity analysis of the uncertainty around the estimated weighted mean disability weight*

The disability weight associated with epilepsy depends on the disease severity (see table 3 in main manuscript). We calculated a weighted mean disability weight for epilepsy across the different severity levels using clinical data of epilepsy patients in an onchocerciasis hyperendemic area [7]. However, as our weighted mean disability weight for epilepsy in our analysis is based on a specific epilepsy population within a specific locality in the Littoral Province of Cameroon, we have performed a sensitivity analysis to assess the uncertainty of our estimated weighted mean disability weight for epilepsy. To assess the uncertainty, we have re-calculated YLDs due to OAE using the weighted mean disability weight as reported by the Global Burden of Disease (GBD) Study (2015) [8]. The YLDs due to epilepsy as reported by the GBD (2015) were estimated to be 1.3 million which was divided by the total estimated number of prevalent epilepsy cases (3.4 million) for the same year in SSA, giving a GBD weighted mean disability weight of 0.380. The results of the sensitivity analysis using the GBD weighted mean disability weight are provided in **Table S4**.

**Table S4. Results of the sensitivity analysis to assess the impact of the applied weighted mean disability weight on the estimated YLDs. Number of cases reported in thousands [95%CI].**

|  | | **Areas where presence of OAE is reported or suspected** | **Areas where presence of OAE has not yet been investigated** |
| --- | --- | --- | --- |
| **Weighted mean disability weight** | | | |
|  | **0.336 (baseline)** | 39 [17 - 148] | 89 [37 - 402] |
|  | **0.380 (GBD)** | 44 [19 - 168] | 100 [41 - 454] |

## *3. Probabilistic sensitivity analysis for calculating YLDs attributable to OAE in 2015*

A weighted mean disability weight for epilepsy can be calculated by multiplying the proportion of epilepsy cases in a given epilepsy severity state by the disability weight for that severity state (as provided by the GBD studies) and summing the products over all severity states. One thousand unique, random draws of the value of each applicable disability weight were sampled from a logit-normal distribution described by the mean and confidence interval bounds for that disability weight as reported by the GBD. Five hundred of these draws were sampled with a standard deviation that was calculated using the lower bound of the confidence interval and 500 were sampled with the standard deviation calculated using the upper bound of the confidence interval since no standard deviation was reported. These calculations were done as follows:

$\frac{[Mean- Lower bound 95\%CI]}{1.96}$ and $\frac{\left[ Upper bound 95\%CI \right]-Mean}{1.96}$

The weighted mean disability weight was then repeatedly calculated using the draws for one disability weight of interest at a time (severe epilepsy, less severe epilepsy and seizure-free/treated epilepsy each done independently) while keeping the other disability weight values constant as their means. This produced three collections of disability weights, each with one disability weight value varying and the other two remaining constant. The disability weight values in each collection were multiplied by their respective proportional contribution to the final disability weight and they were summed. The weighted mean disability weight for each of the three collections was multiplied by the estimated number of prevalent cases to calculate YLDs. The results of the tests of the three disability weights are graphed together, independently for areas with reported or suspected OAE cases, and areas where OAE has not yet been investigated (**Figure S1**).

We additionally performed a multi-way sensitivity analysis with a similar process as described above. Rather than varying the disability weight values one at a time, 1000 draws were drawn from each disability weight distribution independently at the same time – 500 using the standard deviation calculated using the upper bound of the confidence interval and 500 using the standard deviation calculated using the lower bound of the confidence interval. These were multiplied by their respective proportional contribution to the final disability weight and then summed across to get the weighted mean disability weight (**Figure S2**). In order to calculate the YLDs, we then multiplied the estimated weighted mean disability weight by the estimated number of prevalence cases.

**Figure S1.** **Box-and-whisker plots showing the results of a multiple one-way probabilistic sensitivity analysis around the uncertainty of the weighted mean disability weight as applied to calculate the YLDs attributable to OAE by 2015**. Each box represents the mean and interquartile ranges (IQR) of the YLDs stratified by area of interest. The black vertical line are the lower and upper bounds of the 95%CI around the YLDs attributable to OAE. The red dots are outliers as drawn from the logit-normal distribution for each of the disability weights by severity.

**Figure S2. Box-and-whisker plot showing the results of a multi-way probabilistic sensitivity analysis around the uncertainty of the weighted mean disability weight, sampled independently.** The results of the sampled weighted mean disability weight were then applied to calculate the YLDs attributable to OAE by 2015. Each box represents the mean and IQR of the YLDs stratified by area of interest. The black vertical line are the lower and upper bounds of the 95%CI around the YLDs attributable to OAE. The red dots are outliers as drawn from the logit-normal distribution for the weighted mean disability weight.

**References**

1. Pion SDS, Kaiser C, Boutros-Toni F, Cournil A, Taylor MM, Meredith SEO, et al. Epilepsy in onchocerciasis endemic areas: systematic review and meta-analysis of population-based surveys. PLoS Negl Trop Dis. 2009;3:e461.

2. Kamgno J, Pion SDS, Boussinesq M. Demographic impact of epilepsy in Africa: results of a 10-year cohort study in a rural area of Cameroon. Epilepsia. 2003;44:956–63.

3. Shackleton DP, Westendorp RG, Trenité DG, Vandenbroucke JP. Mortality in patients with epilepsy: 40 years of follow up in a Dutch cohort study. J Neurol Neurosurg Psychiatry. 1999;66:636–40.

4. Nilsson L, Tomson T, Farahmand BY, Diwan V, Persson PG. Cause-specific mortality in epilepsy: a cohort study of more than 9,000 patients once hospitalized for epilepsy. Epilepsia. 1997;38:1062–8.

5. Lhatoo SD, Johnson AL, Goodridge DM, MacDonald BK, Sander JW, Shorvon SD. Mortality in epilepsy in the first 11 to 14 years after diagnosis: multivariate analysis of a long-term, prospective, population-based cohort. Ann Neurol. 2001;49:336–44.

6. Ristić AJ, Sokić D V, Trajković G, Janković S, Vojvodić NM, Bascarević V, et al. Long-term survival in patients with status epilepticus: a tertiary referral center study. Epilepsia. 2010;51:57–61.

7. Prischich F, De Rinaldis M, Bruno F, Egeo G, Santori C, Zappaterreno A, et al. High prevalence of epilepsy in a village in the Littoral Province of Cameroon. Epilepsy Res. 2008;82:200–10.

8. GHDx. Global Health Data Exchange. GBD results tool. http//ghdx.healthdata.org/gbd-results-tool. Accessed 12 Dec 2017.
